# Meta-analysis for Epidemiologic Studies on the Relationship between Smoking and Parkinson’s Disease

**DOI:** 10.2188/jea.11.87

**Published:** 2007-11-30

**Authors:** Minoru Sugita, Takashi Izuno, Masayuki Tatemichi, Yumi Otahara

**Affiliations:** Department of Environmental and Occupational Health, Toho University School of Medicine.

**Keywords:** smoking, Parkinson’s disease, epidemiologic study, meta-analysis

## Abstract

Many epidemiologic studies on the relationship between smoking and Parkinson’s disease (PD) have been conducted. Morens et al. reviewed many articles in the study field and concluded that smoking is inversely associated with the risk of PD. In the present study, the object is to obtain summarized risk estimates of the relationship from the published articles using meta-analysis.

Summarized risk estimates on the relationship between smoking and PD were found to be about 0.5 with statistical significance in meta-analysis. Therefore, the result that smoking is inversely associated with the risk of PD is appropriate.

## INTRODUCTION

Many epidemiologic studies on the relationship between smoking and neurologic disorders have been conducted, and Parkinson’s disease (PD) has been sufficiently investigated^1^^-^^63^^)^ in the study field. Morens et al.^47^^)^ published a review article on epidemiologic studies^1^^-^^46^^)^ on the relationship between smoking and PD, and reported that smoking was inversely associated with the risk of PD. However, Morens^47^^)^ did not obtain a summarized risk estimate of the relationship from the published articles^1^^-^^46^^)^ by meta-analysis.

In the present study, we try to obtain the summarized risk estimate and its 95% confidence interval (95%CI) of the relationship from the published articles using meta-analysis. Not only the 46 published articles^1^^-^^46^^)^ cited by Morens^47^^)^ but 16 articles^48^^-^^63^^)^ published after Morens’s study were also included. We discuss whether or not smoking is inversely associated with the risk of PD, using the summarized risk estimate of the relationship calculated in the present study.

## MATERIALS AND METHODS

We collected not only the 46 published articles^1^^-^^46^^)^ which Morens^47^^)^ cited but also many articles published afterward using MEDLINE with smoking, Parkinson’s disease, and epidemiology as key words. If a risk estimate and its 95%CI were not obtained for an article, it was not included in the present study. When two or more articles were published on an identical study, the article in which the relationship between smoking and PD was principally investigated or the article which was published last was selected and the other articles not used. Thirty articles from the 46 articles^1^^-^^46^^)^ which Morens^47^^)^ cited and 16 articles^48^^-^^63^^)^ from the many articles by MEDLINE were selected for calculation of the summarized risk estimates and their 95%CIs for epidemiologic studies on the relationship between smoking and PD.

### 1. Risk estimate and its 95%CI

When the risk estimate and its 95%CI are described in an article, they are employed in the present study. If the risk estimate and its 95%CI are not shown in an article but can be calculated from the contents of the article using the method described below, the values calculated are used in the present study. If the risk estimate and its 95%CI are not given in an article and the values could not be calculated from the contents of the article by any means, the article was removed from the study.

#### 1.1 Univariate analysis

When a risk estimate adjusted by confounding variables as a multivariate analysis with its 95%CI was not described in an article and the adjusted values could not be calculated from the contents of the article, we attempted to obtain the values using univariate analysis, i.e. we calculated crude values unadjusted by confounding variables from the contents. If we were able to make a 2 × 2 contingency table for the relationship between smoking and PD from the contents of the article, the odds ratio and its 95%CI were calculated from the 2 × 2 table in the following manner. Let *a*, *b*, *c*, and *d* be numbers of subjects for smoking (+) and PD (+), smoking (+) and PD (-), smoking (-) and PD (+), and smoking (-) and PD (-) in the 2 × 2 table, respectively. The odds ratio *R* and its 95%CI *R_U_* and *R_L_* are obtained^64^^)^ as

R=ad/(bc), [1]

$$R_{U},R_{L}=R\cdot \exp(\pm 1.96\cdot \sqrt{1/a+1/b+1/c+1/d}).$$ [2]

Other risk estimates and their 95%CIs than odds ratio can be calculated analogously.

#### 1.2 Standardized mortality ratio (SMR)

When SMR can be obtained from the contents of the article, SMR was calculated as the risk estimate. Its 95%CI was calculated approximately from the number of subjects and the mortality rate of PD using Equation [2].

#### 1.3 Multivariate analysis

When both risk estimates with their 95%CIs adjusted and unadjusted by confounding variables are shown in an article, we adopted adjusted values. If the 95%CI is not found but the risk estimate *R* and chi-square test statistic *X*^2^ adjusted by confounding variables are found, using Mantel-Haenszel’s method, the 95%CI *R_U_* and *R_L_* are calculated^65^^)^ to be

$$R_{U},R_{L}=R\cdot \exp(\pm 1.96\cdot \ln R/\sqrt{X^{2}}),$$ [3]

where the degree of freedom for *X*^2^ is 1. When *X*^2^ as multivariate analysis was not obtained, the values were calculated from the simple 2 × 2 table using the above methods.

#### 1.4 Matching methods

In pair matching, let *U* be the number of pairs for smoking (+) in subjects and smoking (-) in controls and *V* be the number of pairs for smoking (-) in subjects and smoking (+) in controls. When *U* and *V* are obtained in an article, using McNemar’S method, the odds ratio *R* and its 95%CI *R_U_* and *R_L_* are obtained^64^^)^ as

R=U/V, [4]

$$\begin{array}{rl} R_{U},R_{L} & =\{1.96^{2}(U+V)+2\cdot U\cdot V\pm 1.96\sqrt{1.96^{2}(U+V{)}^{2}+4\cdot U\cdot V(U+V)}\}\\ & \;/(2\cdot V^{2}). \end{array}$$ [5]

If *U* and *V* were not obtained from an article using a matching method, the values were calculated from an ordinary 2 × 2 table using the above method.

### 2. Meta-analysis

All risk estimates in the present study are indicators which express the ratio of risk between smokers and nonsmokers, e.g. odds ratio. Therefore, we considered all risk estimates to be the same indicators in the present study.

Summarized risk estimates and their 95%CIs were calculated using the method of Fleiss’s paper^66^^)^. When risk estimates for meta-analysis are homogeneous, i.e. the chi-square value for homogeneity test is less than the degree of freedom, a fixed-effects model was used, and in other cases the random-effects model by DerSimonian^66^^, ^^67^^)^ was used. When risk estimates for two or more subgroups were shown in an article but the risk estimate for all subjects was not shown, the risk estimate for all subjects was calculated using Fleiss’s method^66^^)^.

### 3. Comparison between two groups of studies

Statistical tests for the difference between summarized risk estimates of two study groups were conducted assuming the logarithm of risk estimate to be distributed normally. The four sets of the two study groups compared were (1) studies for male and female subjects, (2) prospective and other types of studies, (3) studies published before 1991 and from 1991 to 2000, and (4) studies whose risk estimates were calculated in the present study using Equation [1] and studies whose risk estimates were not calculated in the present study. Risk estimates for only male subjects were available in five studies. These were added to the 41 studies that indicated both genders, and the risk estimates of 46 studies were used, excluding comparison studies for male and female subjects.

### 4. Funnel plot graph

In order to check for publication bias a funnel plot graph^69^^)^ for relationship between the risk estimates of the 46 articles and the standard error of natural logarithm of the risk estimates was drawn.

## RESULTS

Unnecessary articles were removed from the table in the article by Morens^47^^)^, and the 16 articles^48^^-^^63^^)^ published after Morens’s study were added. Table 1 shows risk estimates and their 95%CIs of articles for epidemiologic studies on the relationship between smoking and PD by gender. In Table 1, calculated summarized risk estimates and their 95%CIs by meta-analysis in males, females, and both sexes are shown to be 0.45 (0.36-0.57), 0.53 (0.36-0.78), and 0.57 (0.52-0.63), respectively. Methods of calculation from the contents of the articles in the present study are indicated in Table 1. When risk estimates and their 95%CIs are described in the articles and the values are employed in the present study, calculation methods of the articles are not shown in Table 1.

**Table 1.** Risk estimates and their 95% confidence intervals (95%CI) of epidemiologic studies on relationship between smoking and parkinson’s disease with summarized risk estimates.

Ref No.	Study type	Risk- Est	Calculation method		Risk estimates (95%CIs)		

			Risk-Est	95%CI	Male	Female	All
3	Pros	SMR		Eq[2]	0.23(0.13-0.39)		
4	Pros	SMR	SMR	Eq[2]			0.79(0.54-1.15)
5	CCS	OR		Eq[2]	0.44(0.27-0.71)		
6	CCS	OR		Eq[2]	0.65(0.41-1.04)	0.68(0.45-1.02)	0.66(0.51-0.86)
8	CCS	POR		Eq[3]	0.38(0.22-0.65)	0.56(0.30-1.06)	0.45(0.30-0.68)
9	CCS	OR	2 × 2-CT	Eq[2]	0.22(0.07-0.68)		
10	Pros	SMR	SMR	Eq[2]	0.43(0.18-1.01)		
15	CCS	OR		Eq[5]			0.60(0.16-2.27)
18	CCS	OR		Eq[2]	0.68(0.44-1.06)	0.19(0.10-0.36)	0.46(0.33-0.63)
19	CCS	OR	2 × 2-CT	Eq[2]			0.74(0.55-0.99)
20	CCS	OR	2 × 2-CT	Eq[2]			0.44(0.19-1.02)
21	CCS	OR		Eq[5]	0.53(0.33-0.84)	0.50(0.29-0.87)	0.52(0.36-0.74)
22	CCS	OR		Eq[2]	0.32(0.14-0.71)	0.62(0.27-1.43)	0.44(0.23-0.85)
25	CCS	OR					0.73(0.45-1.17)
26	CCS	OR	2 × 2-CT	Eq[2]			0.44(0.26-0.76)
27	Pros	OR		Eq[3]			0.41(0.24-0.70)
30	CCS	OR		Eq[3]			0.20(0.10-0.38)
32	Prev	POR	2 × 2-CT	Eq[2]	0.95(0.46-1.95)	0.31(0.04-2.34)	0.92(0.49-1.72)
33	CCS	OR		Eq[2]			0.56(0.25-1.24)
34	CCS	OR		Eq[2]	0.8 (0.4 -2.0 )		0.62(0.36-1.06)
35	CCS	OR					0.61(0.18-2.03)
36	CCS	OR		Eq[3]			0.46(0.22-0.95)
37	CCSn	OR					0.70(0.28-1.74)
38	CCS	OR					0.5 (0.3 -0.9 )
39	CCS	OR	2 × 2-CT	Eq[2]			0.57(0.19-1.71)
41	CCS	OR	2 × 2-CT	Eq[2]			0.71(0.43-1.18)
42	CCS	OR	2 × 2-CT	Eq[2]			0.29(0.03-3.13)
43	CCS	OR	2 × 2-CT	Eq[2]	0.26(0.14-0.47)	0.88(0.26-2.99)	0.45(0.28-0.72)
44	CCS	OR					0.32(0.15-0.67)
46	Pros	RR			0.39(0.22-0.70)		
48	CCS	OR					0.85(0.54-1.36)
49	CCS	OR					0.8 (0.4 -1.5 )
50	CCS	OR					0.37(0.18-0.73)
51	CCS	OR			0.47(0.21-1.06)	1.27(0.40-4.00)	0.69(0.38-1.27)
52	CCR	OR					0.50(0.28-0.93)
53	CCS	OR					0.48(0.29-0.80)
54	CCS	OR					0.36(0.17-0.73)
55	CCS	OR					0.5 (0.3 -0.7 )
56	CCS	OR					0.42(0.25-0.70)
57	CCS	OR					1.1 (0.7 -1.8 )
58	CCS	OR	Meta-A	Meta-A			0.73(0.43-1.26)
59	CCS	OR					0.7 (0.4 -1.1 )
60	CCS	OR					0.43(0.23-0.79)
61	CCS	OR	Meta-A	Meta-A			0.57(0.34-0.96)
62	CCS	OR	Meta-A	Meta-A			0.79(0.51-1.24)
63	CCS	OR					0.50(0.29-0.87)

Summarized risk estimates					0.45(0.36-0.57)	0.53(0.36-0.78)	0.57(0.52-0.63)
p-value of homogeneity test					0.031	0.041	0.082

2 × 2-CT: 2 × 2 contingency table,

Calculation method: Calculation method in the present study,

CCS: Case-control study, CCSn: Nested case-control study, Eq: Equation,

Meta-A: Meat-analysis, OR: Odds ratio, POR: Prevalence exposure odds ratio,

Prev: Prevalence study, Pros: Prospective study, Ref No.: Reference number,

Risk-Est: Risk estimate, RR: Relative risk, SMR: Standardized mortality ratio

Table 2 indicates the summarized risk estimates and their 95%CIs of (1) studies for male and female subjects, (2) prospective and other types of studies, (3) studies published before 1991 and from 1991 to 2000, and (4) studies whose risk estimates were calculated in the present study using Equation [1] and studies whose risk estimates were not calculated, and for all studies. Statistical tests for the differences between the summarized risk estimates of the two study groups compared in the four sets are not indicated to be statistically significant in Table 2.

**Table 2.** Summarized risk estimates and their 95% confidence intervals according to (1) gender, (2) study type, (3) published year, and (4) calculation of risk estimate from 2 × 2 contingency table in the present study and p-value of statistical test between the two study groups.

(1)	Gender		

	Male	Female	Male and female
Summarized risk estimates	0.45(0.36-0.57)	0.53(0.36-0.78)	0.47(0.39-0.57)
Number of studies	14	8	22
p-value of homogeneity test	0.031	0.041	0.009
p-value of test for 2 groups	0.72		



(2)	Study type		

	Prospective	Other types	All studies

Summarized risk estimates	0.43(0.27-0.68)	0.56(0.51-0.62)	0.54(0.49-0.60)
Number of studies	5	41	46
p-value of homogeneity test	0.006	0.088	0.012
p-value of test for 2 groups	0.58		



(3)	Published year		

	-1990	1991+	All studies

Summarized risk estimates	0.51(0.44-0.60)	0.57(0.50-0.65)	0.54(0.49-0.60)
Number of studies	23	23	46
p-value of homogeneity test	0.009	0.184	0.012
p-value of test for 2 groups	0.60		



(4)	Calculation from 2 × 2 table in the present study		

	(+)	(-)	All studies

Summarized risk estimates	0.58(0.49-0.74)	0.53(0.48-0.60)	0.54(0.49-0.60)
Number of studies	9	37	46
p-value of homogeneity test	0.216	0.014	0.012
p-value of test for 2 groups	0.75		

In Tables 1 and 2 p-values of homogeneity tests^66^^)^ for the risk estimates are shown, and number of the statistically significant tests was not small. The null hypothesis of the test is that all risk estimates are equal to each other. All of the summarized risk estimates were calculated using the random-effects model by DerSimonian^66^^, ^^67^^)^, because all of the chi-square values for homogeneity test were greater than the degrees of freedom. The numerals of the right row in Table 1 show the values of all subjects including males and females in 41 studies, and the numerals of the right row in Table 2 show the values for the sums of the two groups.

**Figure 1.**
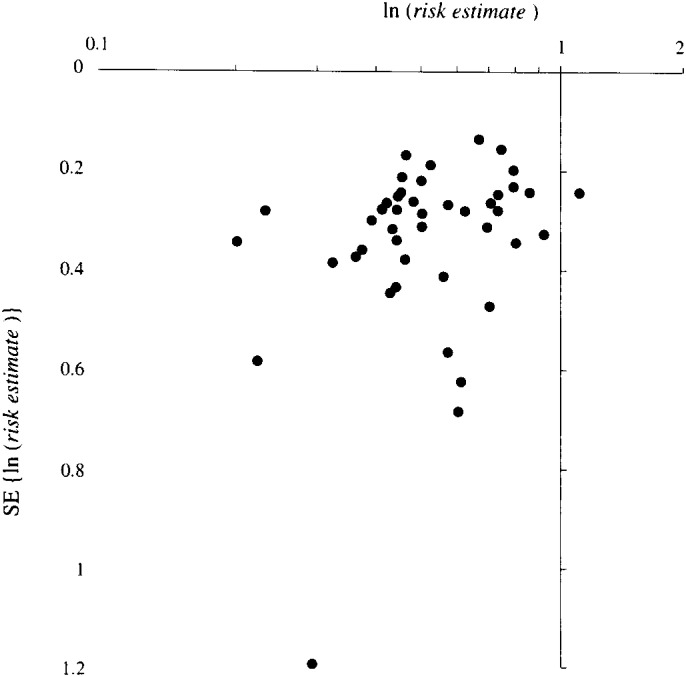
Funnel plot graph for relationship between the risk estimates of the 46 articles and the standard errors of natural logarithm of the risk estimates.

Figure 1 indicates a funnel plot graph^69^^)^ for relationship between the risk estimates of the 46 articles and the standard errors of natural logarithm of the risk estimates. In Figure 1 a few articles with small risk estimates or large standard errors are shown. The summarized risk estimate and its 95%CI was calculated to be 0.57 (0.53-0.62) from the values of the articles removing four articles^3^^, ^^9^^, ^^30^^, ^^42^^)^ whose risk estimates were less than 0.3. The homogeneity test for the risk estimates was not statistically significant, but the chi-square value was greater than the degree of freedom. The summarized risk estimate and its 95%CI was calculated to be 0.54 (0.49-0.60) removing five articles^9^^, ^^15^^, ^^35^^, ^^39^^, ^^42^^)^ whose standard error of natural logarithm of the risk estimates were greater than 0.5, and the homogeneity test for the risk estimates was statistically significant.

## DISCUSSION

The summarized risk estimates between smoking and PD were found to be about 0.5, and all the summarized risk estimates by subgroup were statistically significant (Tables 1 and 2). This means that smoking is inversely associated with the risk of PD, coinciding with the result in the article by Morens^47^^)^.

Morens^47^^)^ decided against attempting a meta-analysis because raw data were not available, the different types of studies hindered data pooling, and some of the data had been adjusted in such a way that “unadjustment” to achieve inter-study comparability of data was impossible. In this study, however, we calculated the summarized risk estimates between smoking and PD using meta-analysis. When many articles are collected for meta-analysis, random error in calculating a summarized risk estimate diminishes because of the law of large numbers. Unsystematic biases can be regarded as random errors. We intended to obtain the summarized risk estimates as qualitative indicators, often used in science as a convenient tool.

Morens^47^^)^ noted that biases in epidemiologic studies on the relationship between smoking and PD were important, and the result that smoking is inversely associated with the risk of PD was criticized because of systematic biases in some articles. The biases are diagnostic displacement, selective mortality, cause-and-effect bias, PD-associated personality differences, smoking-associated symptom/sign suppression, and confounding^47^^)^. Selective mortality, cause-and-effect bias, and smoking-associated symptom/sign suppression are especially regarded as systematic biases in epidemiologic study. It is usually noted that systematic biases affect more results in case-control studies than in cohort studies. In Table 2-(2), the summarized risk estimate in prospective studies is shown to be nearly equal to the value in the other types of studies. This means that systematic biases are not notable in epidemiologic studies on the relationship between smoking and PD. Therefore it is probable that smoking is inversely associated with the risk of PD.

When a risk estimate adjusted by confounding variables as a multivariate analysis with its 95%CI was not described in an article and the adjusted values could not be calculated from the contents of the article, we attempted to obtain the values using univariate analysis, i.e. we calculated crude values unadjusted by confounding variables from the contents using a 2 × 2 contingency table for the relationship between smoking and PD. These 9 articles are indicated in Table 1 as “2 × 2-CT.” In Table 2-(4) the difference between the summarized risk estimate of the 9 and that of the other articles is shown to be small. Therefore the unadjusted values do not contain large biases ignoring confounding factors. In the 9 articles sampling methods with due consideration for bias reduction were described.

The funnel plot graph (Figure 1) indicates a few articles with small risk estimates or large standard errors. However, the differences between the summarized risk estimates with and without these values were not notable. Therefore it may safely be said that publication bias in the study area between smoking and PD is not a serious problem. Some papers^70^^-^^72^^)^ reported that publication biases did not significantly affect results in medical study areas.

When a risk estimate and its 95%CI were not given in an article and the values could not be calculated from the contents of the article by any means, the article was removed from the study. In the present study we employed risk estimates and their 95%CIs if they could be obtained in some way. The employed values may include large biases. However, statistical tests for the differences between the summarized risk estimates of the two study groups compared in the four sets are not shown to be significant in Table 2. The sensitivity analyses in Table 2 and the funnel plot graph for inquiry about publication bias do not indicate the results in the present study to be affected by severe biases.

All of the chi-square values for homogeneity tests of risk estimates were greater than the degrees of freedom, and some of the statistical tests were significant. This implies that risk estimate in the epidemiologic study area between smoking and PD is not distributed in a narrow range. On the other hand, odds ratio^71^^)^ between passive smoking and lung cancer is distributed in a narrow range. Difficulty in the diagnosis of PD may be a cause of this difference.

In the present study misclassification of smoking and diagnosis of PD was not considered. Non-differential misclassification leads to the possibility of underestimating an effect^68^^)^. The summarized risk estimate calculated in the present study may be underestimated, and the true value may be less than the calculated value. Therefore we can conclude with even more probability that smoking is inversely associated with the risk of PD.

The result in the present study does not mean that smoking is recommended because of the inverse association with the risk of PD. Many epidemiologic studies have revealed that smoking is a significant cause of major diseases, e.g. lung cancer. The result in the present study suggests that effective drugs for PD can be developed using chemical substances derived from cigarette smoke.

We could not consider the quality level of the articles^3^^-^^46^^, ^^48^^-^^63^^)^ used in the present study because of the different types of studies and various types of differential misclassification. However, this does not mean that the result in the present study is not valid, because the summarized risk estimate obtained is located far from zero and statistically significant.
